# Peanut Allergy, Allergen Composition, and Methods of Reducing Allergenicity: A Review

**DOI:** 10.1155/2013/909140

**Published:** 2013-07-21

**Authors:** Yang Zhou, Jin-shui Wang, Xiao-jia Yang, Dan-hua Lin, Yun-fang Gao, Yin-jie Su, Sen Yang, Yan-jie Zhang, Jing-jing Zheng

**Affiliations:** ^1^College of Food Science and Technology, Henan University of Technology, Zhengzhou 450001, China; ^2^College of Bioengineering, Henan University of Technology, Zhengzhou 450001, China

## Abstract

Peanut allergy affects 1-2% of the world's population. It is dangerous, and usually lifelong, and it greatly decreases the life quality of peanut-allergic individuals and their families. In a word, peanut allergy has become a major health concern worldwide. Thirteen peanut allergens are identified, and they are briefly introduced in this paper. Although there is no feasible solution to peanut allergy at present, many methods have shown great promise. This paper reviews methods of reducing peanut allergenicity, including physical methods (heat and pressure, PUV), chemical methods (tannic acid and magnetic beads), and biological methods (conventional breeding, irradiation breeding, genetic engineering, enzymatic treatment, and fermentation).

## 1. Introduction

Food allergy is a worldwide health problem. It affects approximately 5% of young children and 3% to 4% of adults in westernized countries [1], and it becomes more and more common in developing countries. Although virtually any food can cause allergy, over 90% of food allergy is triggered by eight food sources: milk, egg, peanut, tree nuts, shellfish, fish, wheat, and soy [2]. Among them, peanut is one of the most allergenic. Peanut allergy affects many individuals and its prevalence is increasing rapidly (the prevalence of peanut allergy in some countries is summarized in Table 1). In western countries, the prevalence of peanut allergy in children in the USA increased from 0.4% in 1997 to 1.4% in 2008 [3]; the prevalence of sensitization to peanuts of 3-year olds in the UK rose from 1.3% to 3.2% between 1989 and 1995 [4]; over 1% of Canadian children are allergic to peanuts [5]; the prevalence of peanut allergy in Denmark and France is 0.2–0.4% and 0.3–0.75%, respectively [6, 7]. In Asia, although few epidemic studies of peanut allergy have been carried out, a study suggests that 0.47% of 14–16-year-old local Singapore schoolchildren and 0.43% of 14–16-year-old Philippine schoolchildren are allergic to peanuts [8]. Considering that 76.8% of Singapore residents are Chinese [9], peanut allergy is likely to become serious in China in the future. Moreover, peanut allergy can sometimes be life-threatening and usually cannot be outgrown, and it is almost impossible to avoid accidental ingestion [10, 11]. Therefore, peanut allergy greatly reduces the life quality of the patient [12] and brings trouble to food industry in allergen labeling. Solving this problem has a great significance not only to the peanut-allergic individuals but also to the food industry. 

## 2. Peanut Allergen

To date, 13 peanut allergens (Ara h 1 through h 13) have been recognized by the Allergen Nomenclature Sub-Committee of the International Union of Immunological Societies. These allergens come from 7 protein families. Except for Ara h 1 (150 kD) and Ara h 3 (360–380 kD), the molecular weight of the other allergens ranges from 5 to 17 kD [13]. The genes corresponding to the 13 allergens have already been elucidated, the sequence of many linear epitopes of peanut allergens has been identified (Table 2), and the 3D models of Ara h 1-Ara h 6 have been built. Although the allergenicity of these allergens has not been thoroughly studied and there is still some debate about the definition of major allergens, the major peanut allergens that are most widely accepted are Ara h 1, Ara h 2, and Ara h 3. 

### 2.1. Ara h 1

**Figure 1:**
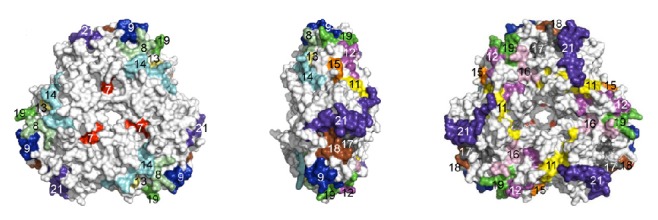
IgE epitopes are mapped on the surface of the 3D model of Ara h 1 core region [16].

Ara h 1 is a glycoprotein and belongs to the vicilin (7S) family. It comprises 12–16% of the total peanut protein [14] and affects 35–95% of peanut-allergic patients in different populations [15]. Native Ara h 1 exists as a trimer formed by three identical monomers, and the crystal structure of its core region has been elucidated (Figure 1) [16]. The topology and basic structure of its core region are very similar to other known structures of 7S globulins. Those similarities indicate that there is a high possibility of cross-reactivity between 7S globulins [16]. To date, 21 linear epitopes have been identified on the mature Ara h 1 [17–20], and 14 epitopes were found in the core region [16]. It is found that most epitopes on the core region become either slightly (<50% burial) or significantly (≥50% burial) buried upon trimer formation [16]. The burial of those epitopes likely explains the relatively weak activity of native (trimer) Ara h 1 in cross-linking IgE and the strong binding of IgE to denatured monomers [18, 21].

### 2.2. Ara h 2

**Figure 2:**
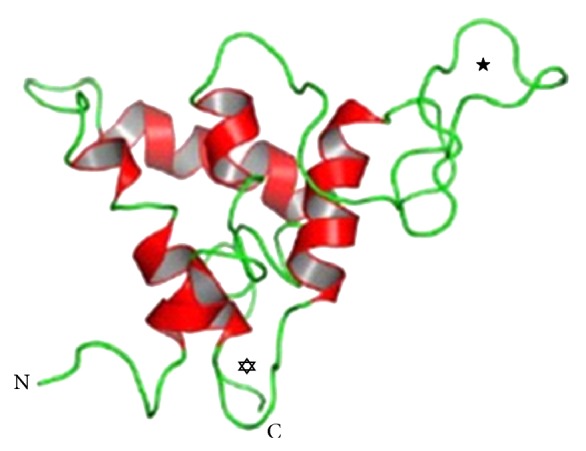
Ribbon diagram of Ara h 2 [26].

Ara h 2 (16-17 kDa) is also a glycoprotein and accounts for 5.9–9.3% of the total peanut protein [22]. It is a 2S albumin, also known as conglutin, and functions as a trypsin inhibitor [23]. More than 95% of peanut-allergic individuals in the USA have specific IgE to Ara h 2, and Ara h 2 was found to be a more potent allergen than Ara h 1 [21, 24, 25]. The structure of Ara h 2 is five *α*-helices arranged in a right-handed superhelix and connected by several extended loops (Figure 2). This three-dimensional conformation is stabilized by four conserved disulphide bridges. Ten epitopes have been mapped on Ara h 2, and these epitopes show a fairly well exposition on the molecular surface [26].

### 2.3. Ara h 3

**Figure 3:**
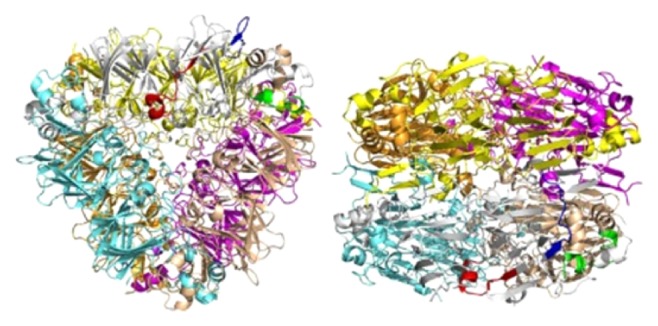
Ara h 3 is represented with each of the monomers shown in a different color. In the gray monomer, linear epitope 1, 2, and 3 are shown in red, green, and blue, respectively [30].

Ara h 3 is a seed storage protein and belongs to the legumin (11S) family [27]. It is recognized by 50% of peanut-allergic individuals and also functions as a trypsin inhibitor [28, 29]. Ara h 3 and soybean glycinin result in a sequence identity of 47.2% [30]. Mature Ara h 3 is a hexamer (360–380 kD) formed by a head-to-head association of two trimers (Figure 3) [30]. Each monomer was found to have 4 linear epitopes [31]. In the natural form of Ara h 3, epitope 4 is fully exposed, while the side chains of most of the critical residues of the other three epitopes are completely or nearly completely buried. This suggests that linear epitopes 1 and 2 may not be recognized by IgE antibodies in the intact form, while epitope 4 and part of epitope 3 may be allergic in the natural form of Ara h 3 [30]. 

### 2.4. Ara h 4

Ara h 4 is actually an isoform of Ara h 3. Now, it is no longer thought to be a distinct allergen and renamed to Ara h 3.02 [13, 32]. 

### 2.5. Ara h 5

**Figure 4:**
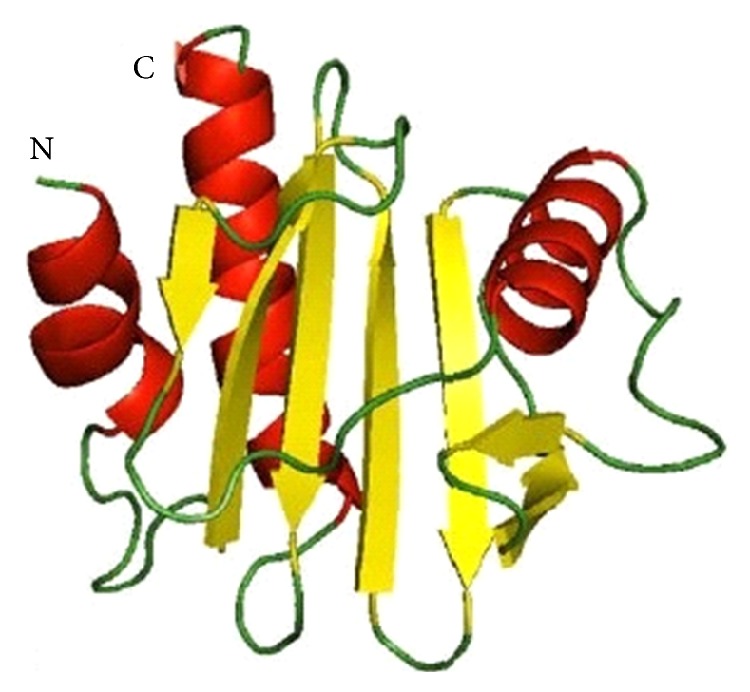
Ribbon diagram of the three-dimensional model of Ara h 5. Strands of b-sheet and stretches of a-helix are in yellow and red, respectively. Coil structures or loops are in green, N and C indicate the N- and C-terminus of the polypeptide, respectively [38].

Ara h 5 (15 kD) belongs to the profilin family and regulates the polymerization of actin [13, 32]. It is presented at low levels in peanut extracts and is recognized by 13% of 40 patients' sera [27, 33]. The structure of Ara h 5 is shown in Figure 4.

### 2.6. Ara h 6

**Figure 5:**
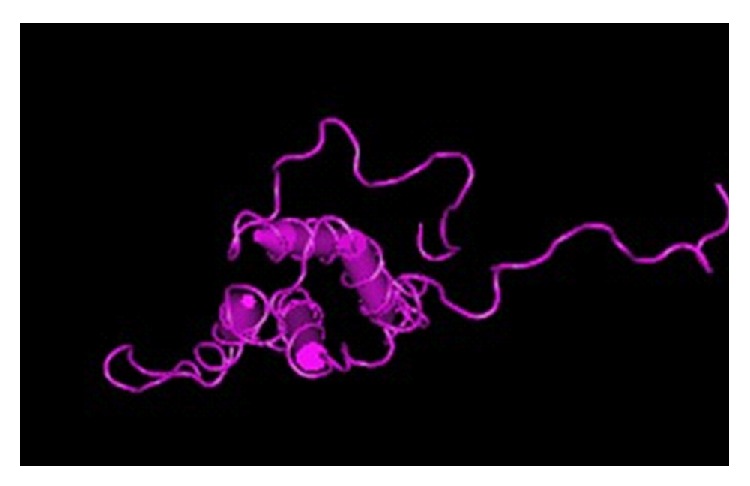
Ara h 6 (PDB Entry 1W2Q, first molecule in the entry).

Ara h 6 is a 15 kD protein and belongs to the conglutin family [13]. It is 59% homologous to Ara h 2 and has similar allergenicity [34, 35]. Ara h 6 is a heat and digestion stable protein and showed resistance to proteolytic treatment [36, 37]. The structure of Ara h 6 is shown in Figure 5.

### 2.7. Ara h 7

Ara h 7 is also a 15 kD protein and belongs to the conglutin family [13]. The sequence identity between Ara h 2 and Ara h 6 is 35%, and it is recognized by 13% of 40 patients' sera [23].

### 2.8. Other Peanut Allergens

Ara h 8 (17 kD) is a Pathogenesis-related protein. Ara h 9 (9.8 kD, 2 isoforms) is a nonspecific lipid-transfer protein. Ara h 10 (16 kD, 2 isoforms) and Ara h 11 (14 kD) belong to oleosin. Ara h 12 and Ara h 13 are defensin, with molecular weight ranging from 5 to 12 kD [13]. 

## 3. Harm of Peanut Allergy 

Peanut-allergic reactions involve the skin, the respiratory tract, and the gastrointestinal tract [39]. The common symptoms include acute urticaria, acute vomiting, laryngeal oedema, hypotension, and dysrhythmia [40, 41]. Peanut allergy is very dangerous. Ingestion of even a trace amount of peanut may elicit life-threatening reactions within minutes [42]. Peanut, together with tree nuts, causes most of the fatal or near-fatal food-related anaphylaxis, and peanut allergy leads to 100–200 deaths each year in the USA [43, 44]. Moreover, peanut allergy is usually lifelong, with only 10% of peanut-allergic children outgrowing it [10]. Last but not least, due to the ubiquitous use of peanut in food industry, it is almost impossible for a peanut-allergic patient to completely avoid peanut even if he/she strictly obeys the doctor's guidance. Studies suggest that up to 75% of individuals with known peanut allergy experience reactions caused by accidental exposure [11]. Thus, peanut allergy gives enormous pressure to the patients and their families and greatly impairs their life quality [12]. In addition, the US law demands that allergen content be labeled on any product sold in the USA [45], and tracing and determining peanut allergens in food products increase the cost and bring inconvenience to the food trade.

## 4. Methods of Reducing Allergenicity

Although there is now no feasible solution to peanut allergy, many methods have shown great prospect, including oral immunotherapy and some methods to reduce peanut allergenicity. The methods of reducing peanut allergic potential are reviewed as follows.

### 4.1. Physical Methods

#### 4.1.1. Heat and Pressure Treatment

There are three ways to decrease peanut allergenicity by heat treatment. The first is roasting. Roasting has been recognized as a process that can increase peanut allergenicity [46]. However, Vissers et al. found that after heating Ara h 2/6 (purified from raw peanuts) in a dry form for 20 min at 145°C, the IgE-binding capacity and the degranulation capacity of Ara h 2/6 were 600–700-fold lower than those in the native form [47]. 

The second is boiling. Boiling native Ara h 2/6 (15 min, 110°C) and boiling native Ara h 1 (15 min, 100°C) resulted in decreased IgE reactivity and mediator-releasing capacity; but for Ara h 2/6 and Ara h 1 extracted from roasted peanut, boiling had no effect [48, 49].

The third one is autoclaving. Cabanillas et al. discovered that IgE-binding capacity of peanut allergens is significantly decreased by autoclaving at 2.56 atm, for 30 min [50]. However, this method obviously comes with high energy consumption and expensive devices.

#### 4.1.2. PUV

Pulsed ultraviolet light (PUV) is another effective method in reducing peanut allergenicity. Yang et al. treated protein extracts from raw and roasted peanuts and peanut butter slurry in a Xenon Steripulse XL 3000 PUV system. The treatment time was 2, 4, and 6 min for protein extracts and 1, 2, and 3 min for peanut butter slurry. The distance from the central axis of the lamp was varied at 10.8, 14.6, and 18.2 cm. The research found that PUV treatment resulted in reduction in the level of Ara h 1, Ara h 2, and Ara h 3 and decreased IgE binding ability by 12.9% to 6.7% [51]. However, like all the other irradiation technologies, this method comes with concern of food safety.

### 4.2. Chemical Methods

#### 4.2.1. Tannic Acids

Chung and Reed reduced the allergenicity of peanut butter by adding tannic acid. The principal is that tannic acid interacts with allergens to form indigestible complex, and epitopes on the allergens are covered during complex formation, making the epitopes inaccessible to antibodies and resulting in reduced allergenicity. Chung and Reed added tannic acid to a peanut butter extract (5 mg/mL; pH = 7.2) and discovered that when pH = 2 and pH = 8, the complexes do not release Ara h 1, or Ara h 2, and the IgE binding ability is decreased; and when concentration of tannic acid is 1-2 mg/mL, the IgE binding ability of the complex is reduced substantially [52]. Since tannic acid interacts with both allergen and non-allergen peanut proteins, such treatment has two obvious deficiencies: first, peanut nutrition is reduced to a great extent, and second, intake of much indigestible food may cause stomach discomfort and thus greatly limit consumption of peanut products.

#### 4.2.2. Magnetic Beads

Magnetic beads can also be used to remove peanut allergens. The principle is that phenolic compounds and ferric ions (Fe^3+^) can bind to peanut allergens; thus, one can reduce peanut allergenicity by using magnetic beads attached with or without phenolics to capture peanut allergens or allergen-Fe^3+^ complexes and then separate the beads by a magnetic device. Chung and Champagne found the following: treating peanut extracts by CHL beads (magnetic beads covalently attached with chlorogenic acid, a phenolic) resulted in marked decrease of Ara h 1 and small reduction of Ara h 2; when using magnetic beads without phenolic compounds to treat peanut extracts that have been incubated with Fe^3+^ and dialyzed, both Ara h 1 and Ara h 2 were markedly reduced; those two methods reduced IgE binding ability of the treated extracts by 28–47%. Chung and Champagne believed that the magnetic beads system was a simple way to partially remove peanut allergens from peanut extracts, and it could be a potential approach to produce hypoallergenic peanut products and beverages [53]. 

### 4.3. Biological Methods

#### 4.3.1. Conventional Breeding

The rationale of conventional breeding is crossing hypoallergenic varieties to produce a variety that is more hypoallergenic. Perkins et al. crossbred peanuts that were missing either an Ara h 2 or Ara h 3 isoform and produced a variety lacking both isoforms. The observed numbers of the new variety conformed to the 15 : 1 Mendelian dihybrid ratio [54]. However, considering the large amount of peanut allergens, the progress of this method seems very slow. 

#### 4.3.2. Irradiation Breeding

As for mutation breeding, a type of technology is well worth mentioning. It is the heavy-ion beam irradiation (HIBI). This technology leads to mutation and inactivation of a single gene or multiple genes in a plant, thus inducing stable knockout mutants [55, 56]. Cabanos et al. treated a Japanese peanut variety—Nakateyutaka—with either N or C heavy-ion beams at a dose of 100 Gy and obtained seventeen knockout mutants from 11,335 screened M2 seeds. Among the seventeen mutants, eight lacked either one of the two isoforms of Ara h 2, and the other nine are missing one of the isoforms of Ara h 3 [57]. Cabanos et al. believe that HIBI is a powerful means of producing knockout hypoallergenic peanuts and has many advantages [57], including low radiation exposure levels, less cellular damage, no need for tedious tissue culture or regenerative procedures, no severe growth inhibition, and, in general, less plant death and a high rate of mutation producing diverse kinds of mutants [55, 56, 58]. However, like all the other irradiation technologies, HIBI comes with the concern of food safety. 

#### 4.3.3. Genetic Engineering

Great advance has been made in removing peanut allergens by genetic technology. Chu et al. silenced Ara h 2 and Ara h 6 by RNA interference and produced three independent transgenic lines. All the three lines were featured by significant reduction in human IgE binding to Ara h 2 and Ara h 6 as well as the level of Ara h 2, whereas the level of Ara h 6 was only reduced in two lines. In addition, there were no significant differences between the seed weight and germination data of transgenic and nontransgenic plants [59]. Another research comes from Ananga et al. who tried to produce hypoallergenic peanuts by silencing Ara h 1, Ara h 2, and Ara h 3 with RNA interference. Ananga et al. have found the following: the percentages of transgenic peanut that showed reduction in Ara h 1, Ara h 2, and Ara h 3 were 9%, 10%, and 16%, respectively 3% transgenic seeds were free of all three allergens; the IgE-binding capacity was significantly reduced in at least nine transgenic seeds with reduction in Ara h 1 or Ara h 2, Ara h 3 [60]. Although genetic technology has shown great promise to produce allergen-free peanut, this technology also has two big drawbacks. One is people's increasing repulsion to transgenic food. The other is the fact that peanut allergens account for 20–30% of total peanut proteins, and if all the allergens are removed, peanuts may not taste like peanuts. 

#### 4.3.4. Enzymatic Treatment

Enzymatic treatment is full of potential to produce allergen-free peanut and there are two types of enzymatic treatment. 

One is using enzymes to cross-link allergen proteins, resulting in the burial of epitopes. Chung and Champagne treated protein extract from roasted and raw peanuts with peroxidase (POD) and transglutaminase (TGA) at 37°C. Both enzymes catalyze cross-links between proteins. Chung and Champagne found the following: POD treatment of roasted peanut resulted in partial loss of Ara h 1 and Ara h 2 along with reduced IgE binding ability and formation of new polymers; on the other hand, TGA treatment of roasted peanut had no effect on the content of Ara h 1 and Ara h 2 as well as IgE binding ability; both POD and TGA had no effect on the IgE binding ability of protein extract from raw peanut. Chung and Champagne believed that POD may be useful in desensitizing peanut while TGA should be useless [61]. 

The other is using enzymes to break down allergens, destroying their epitopes. Cabanillas et al. studied the effect of hydrolysis with alcalase and flavourzyme on the allergenicity of the soluble protein fraction of roasted peanut. Parameters for alcalase hydrolysis were *S* = 2%, *E*/*S* = 0.4 AU/g of protein, *T* = 50°C, and pH 8.0; parameters for flavourzyme hydrolysis were *S* = 2%, *E*/*S* = 100 LAPU/g of protein, *T* = 50°C, and pH 7.0. Cabanillas et al. discovered the following: 30 min alcalase treatment resulted in an important decrease of Ara h 1, Ara h 2, and Ara h 3 levels and reduced IgE binding reactivity by 98%; 90 min alcalase treatment could fully eliminate IgE binding reactivity; while 30 min flavourzyme treatment caused an increase in IgE reactivity, hydrolyzing with flavourzyme for 300 min led to a 65% inhibition of IgE reactivity [62]. Although Cabanillas et al. demonstrated that enzymatic treatment with alcalase or flavourzyme could reduce IgE reactivity in peanuts, Guo et al. found that the allergenicity was retained after treating roasted peanut protein extract in a similar way [63]. The two researches adopted different methods to assess the allergenicity of enzymatic products, and the liability of such assessment methods is still in debate. It is unclear whether alcalase and flavourzyme have an effect or not. 

The most promising enzymatic method for desensitizing peanut is the research of Ahmedna et al. Mohamed's team has been working on the subject for over 7 years. They have developed a method which is very likely to completely eliminate peanut allergenicity in a quick, simple, and inexpensive way, without greatly changing the flavor and texture of natural peanuts. Since Mohamed's team are using their research to apply a patent, only a little detail of the method can be obtained. It is only told that peanut allergenicity is reduced by direct application of enzymatic solution to either raw, blanched, or roasted peanuts, or peanut products or derivatives (including but not limited to peanut butter, peanut kernels, peanut skins, peanut protein isolate, peanut flour, or peanut milk); the enzymatic solution used in this method contains at least one endopeptidase whose hypoallergenically effective amount is at least 0.001% (w/w) [64]. 

#### 4.3.5. Fermentation

Few studies have been reported on reducing peanut allergenicity by fermentation. It is only reported that Dr. Ahmedna et al. found that fermenting whole or ground peanuts with an edible fungus reduced the detectable level of major allergenic proteins Ara h 1 and Ara h 2 by as much as 70 percent, and this study is still in the early stages [65]. Although fermentation method has rarely been reported, this method has already successfully reduced the allergic potential of soybean meal and bovine whey proteins [66, 67] and is very likely to reduce the allergenicity of peanuts. The major principle of fermentation is almost the same as that of enzymatic treatment, and fermentation has all the merits of the enzyme method. Furthermore, this method is usually much cheaper. Therefore, fermentation is still a very promising method to produce hypoallergenic peanuts.

## 5. Prospect

Breeding is an effective way, but some problems cannot be ignored. The advance of conventional breeding seems very slow, and mutation breeding is always involved with the problem of food safety; all of these impede the development of the breeding method.

Heat and pressure treatment is another effective approach to reduce peanut allergenicity. However, it still has some deficiencies: roasting can only be an assistant method; the effect of boiling is very limited; autoclaving requires high energy consumption and expensive devices. All of these constitute obstacles to the development of this method.

Transgenic technology is very promising to produce allergen-free variety in the near future. However, with people's repulsion to transgenic food and flavor problems, it has a long way to go to have transgenic allergen-free peanuts in the market. 

Tannic acid is a useful agent, but considering its obvious drawbacks, it can only be an assistant approach. PUV has the same problem of mutation breeding which hinders its development. Magnetic beads capture is a promising way to decrease peanut allergenicity.

At present, enzymatic treatment is the most promising way to produce nonallergic peanuts. Compared with breeding and gene technology, enzymatic treatment is mild, is natural, usually does not produce harmful substance and can be readily accepted by the public. Compared with autoclaving, the approach of Mohamed's team is very cheap. Moreover, enzymatic treatment does not impair peanut nutrition value. Therefore, the authors believe that it is the most promising approach nowadays.

Fermentation is full of potential to reduce peanut allergenicity. Moreover, it has all the merits of the enzyme method and is usually much cheaper. Although this method has rarely been reported, it may be the best way to reduce peanut allergenicity. 

## Figures and Tables

**Table 1:** The prevalence of peanut allergy in some countries [3–8].

Countries	Prevalence
US children	1.40%
Britain children	3.2%
Canadian children	1.03%
Denmark	0.2–0.4%
France	0.3–0.75%
Local Singapore schoolchildren (14–16 years old)	0.47%
Philippine schoolchildren (14–16 years old)	0.43%

**Table 2:** Sequence of linear epitopes of peanut allergens [17, 19, 26, 30].

Allergen	Epitope number	Epitope sequence
Ara h 1 core region	7^a^	PGQFEDFF
	8^a^	YLQGFSRN
	9^a^	FNAEFNEIRR
	10^a^	QEERGQRR
	11^a^	DITNPINLRE
	12^a^	NNFGKLFEVK
	13^a^	GNLELV
	14^a^	RRYTARLKEG
	15^a^	ELHLLGFGIN
	16^a^	HRIFLAGDKD
	17^a^	IDQIEKQAKD
	18^a^	KDLAFPGSGE
	19^a^	KESHFVSARP
	21^b^	NEGVIVKVSKEHVEELTKHAKSVSK

Ara h 2	1	HASARQQWEL
	2	QWELQGDRRC
	3	DRRCQSQLER
	4	LRPCEQHLMQ
	5	KIQR.DEDSYE
	6	YERDPYSPSQ
	7	SQDPYSPSPY
	8	DRLQ..GRQQEQ
	9	KRELRNLPQQ
	10	QRCDLDVESG

Ara h 3	1	IETWNPNNQEFECAG
	2	GNIFSGFTPEFLAQA
	3	VTVRGGLRILSPDRK
	4	DEDEYEYDE–EDRRRG

^a^Determined by [17]. ^b^Determined by [19].
